# Extracellular glucose and dysfunctional insulin receptor signaling independently upregulate arterial smooth muscle TMEM16A expression

**DOI:** 10.1152/ajpcell.00555.2023

**Published:** 2024-03-04

**Authors:** Somasundaram Raghavan, Masuma Akter Brishti, Angelica Bernardelli, Alejandro Mata-Daboin, Jonathan H. Jaggar, M. Dennis Leo

**Affiliations:** ^1^Department of Physiology, University of Tennessee Health Science Center, Memphis, Tennessee, United States; ^2^Department of Pharmaceutical Sciences, University of Tennessee Health Science Center, Memphis, Tennessee, United States

**Keywords:** diabetic vascular disease, insulin receptor, TMEM16A, vascular smooth muscle

## Abstract

Diabetes alters the function of ion channels responsible for regulating arterial smooth muscle membrane potential, resulting in vasoconstriction. Our prior research demonstrated an elevation of TMEM16A in diabetic arteries. Here, we explored the mechanisms involved in Transmembrane protein 16A (*TMEM16A)* gene expression. Our data indicate that a Snail-mediated repressor complex regulates arterial *TMEM16A* gene transcription. Snail expression was reduced in diabetic arteries while TMEM16A expression was upregulated. The *TMEM16A* promoter contained three canonical E-box sites. Electrophoretic mobility and super shift assays revealed that the −154 nt E-box was the binding site of the Snail repressor complex and binding of the repressor complex decreased in diabetic arteries. High glucose induced a biphasic contractile response in pressurized nondiabetic mouse hindlimb arteries incubated ex vivo. Hindlimb arteries incubated in high glucose also showed decreased phospho-protein kinase D1 and TMEM16A expression. In hindlimb arteries from nondiabetic mice, administration of a bolus dose of glucose activated protein kinase D1 signaling to induce Snail degradation. In both in vivo and ex vivo conditions, Snail expression exhibited an inverse relationship with the expression of protein kinase D1 and TMEM16A. In diabetic mouse arteries, phospho-protein kinase D1 increased while Akt2 and pGSK3β levels declined. These results indicate that in nondiabetic mice, high glucose triggers a transient deactivation of the Snail repressor complex to increase arterial TMEM16A expression independently of insulin signaling. Conversely, insulin resistance activates GSK3β signaling and enhances arterial TMEM16A channel expression. These data have uncovered the Snail-mediated regulation of arterial TMEM16A expression and its dysfunction during diabetes.

**NEW & NOTEWORTHY** The calcium-activated chloride channel, TMEM16A, is upregulated in the diabetic vasculature to cause increased vasoconstriction. In this paper, we have uncovered that the *TMEM16A* gene expression is controlled by a Snail-mediated repressor complex that uncouples with both insulin-dependent and -independent pathways to allow for upregulated arterial protein expression thereby causing vasoconstriction. The paper highlights the effect of short- and long-term glucose-induced dysfunction of an ion channel expression as a causative factor in diabetic vascular disease.

## Graphical abstract

**Figure:**
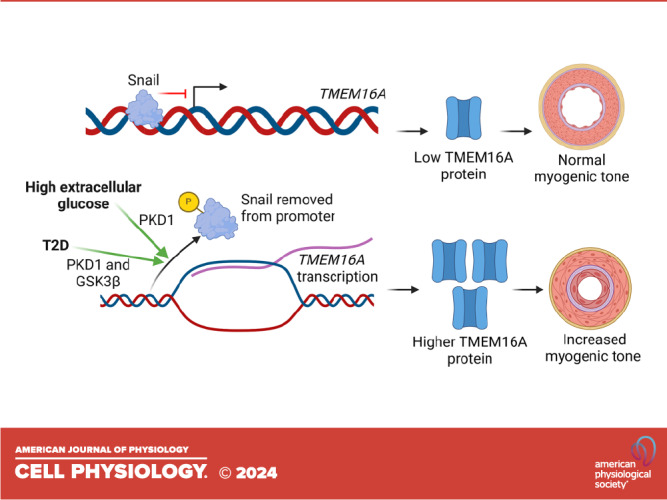

## INTRODUCTION

According to the latest report by the Centers for Disease Control, diabetes affects ∼37.3 million people in the United States with ∼96 million people having “prediabetes” (1). There are two main types of diabetes, type 1 (T1D) or insulin-dependent and type 2 (T2D) or noninsulin-dependent diabetes (2–4). T2D is the most prevalent form affecting ∼90% of patients. Most individuals with T2D concurrently are diagnosed as “insulin-resistant” and usually have obesity, hyperlipidemia, and/or hypertension as comorbidities. This clustering of symptoms is often referred to as “metabolic syndrome” (2–4). Diabetes is also a strong risk factor for peripheral artery disease (PAD), which manifests as lower extremity arterial occlusion and is usually suggestive of wider cardiovascular dysfunction (5, 6). Identifying critical cell-signaling pathways that lead to arterial dysfunction in T2D could pave the way for novel and better therapies to treat cardiovascular diseases (CVD) (2, 4).

The contractile state of smooth muscle cells in the arterial wall of small resistance-size arteries controls regional blood flow and modulates systemic blood pressure. T2D alters the expression and activities of several cation channels that control membrane potential and calcium influx in arterial smooth muscle cells (7). These include the voltage-dependent Ca^2+^ (Ca_V_1.2) channel, large-conductance calcium (Ca^2+^)-activated potassium (K^+^, BK) channel, voltage-dependent K^+^ (K_V_) channel, and several members of the transient receptor potential (TRP) channel family (7, 8). This dysregulation of channel expression causes decreased vasodilation or vasoconstriction. We have previously reported that T2D-induced upregulated expression of TMEM16A, a Cl^−^ channel, in arterial smooth muscle cells, caused increased vasoconstriction in hindlimb arteries of diabetic mice (9). These data demonstrated that dysfunctional Akt signaling in hindlimb arteries was linked to the upregulation of TMEM16A expression during diabetes (9).

Here, we investigated the mechanisms leading to TMEM16A upregulation in diabetic arteries. Using both in vivo and ex vivo approaches, we show that the *TMEM16A* gene promoter region is constitutively occupied by a transcriptional repressor, Snai1 (Snail). High glucose and dysfunctional Akt signaling activate protein kinase D1 (PKD1) and GSK3β, respectively, which negates the repressor effect of Snail on the *TMEM16A* promoter leading to upregulated gene transcription. These data show that both insulin-independent and -dependent pathways can modulate arterial TMEM16A expression.

## METHODS

### Ethics Approval

All experimental protocols were in accordance with institutional guidelines approved by the Institutional Animal Care and Use Committee, UTHSC (Protocol # 22–0354, Most recent reapproval of original protocol: May 2, 2022).

### Animals

**Figure 1.**
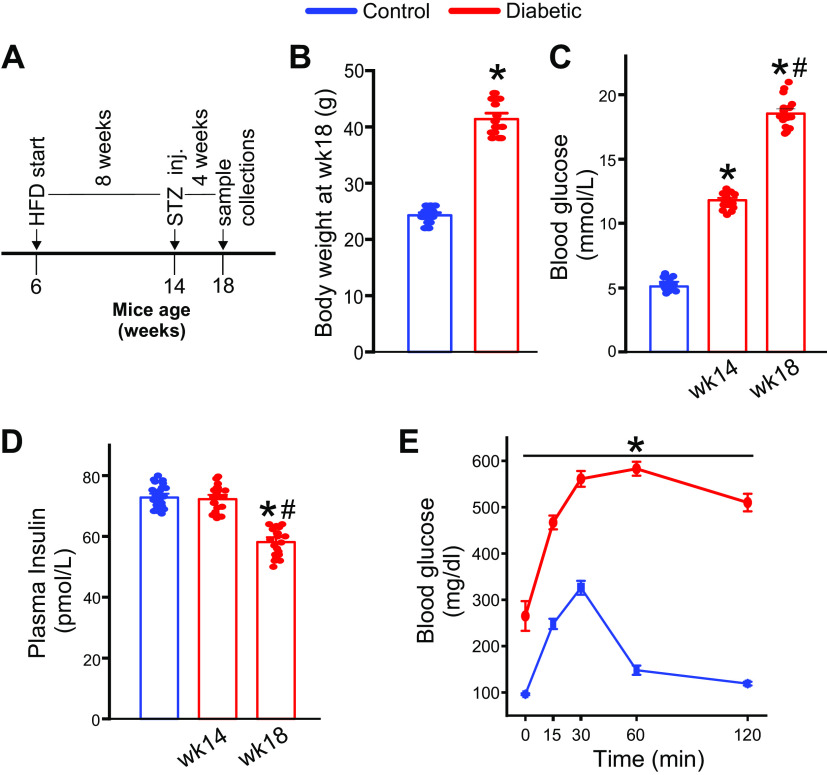
High-fat diet (HFD) with low-dose streptozotocin (STZ) induces type-2 diabetes (T2D) in C57BL6 mice. *A*: schematic of the diet/STZ protocol followed for induction of T2D phenotype. *B*: body weight (in g) recordings from *week 18* mice on HFD after STZ injections compared with age-matched mice treated with regular chow and buffer-alone injections. *n* = 18 for each. *P < 0.05 vs. control. *C*: fasting blood glucose (in mmol/L) recordings from mice before (*week 14*) and after (*week 18*) STZ injections compared with control mice at *week 18*. *n* = 18 each. *P < 0.05 vs. control and #*P* < 0.05 vs. *week 14*. *D*: plasma insulin (in pmol/L) of control (*week 18*) and from mice before (*week 14*) and after (*week 18*) STZ injections. *n* = 18 each. *P < 0.05 vs. control and #*P* < 0.05 vs. *week 14*. *E*: oral glucose tolerance test in control and HFD-STZ mice at *week 18*. *n* = 6 for each. *P < 0.05 vs. control.* n* Values represent number of mice used. Statistical analysis was performed using Student’s *t* test for data in *B* and one-way ANOVA for data in *C-E*.

Male C57BL/6J mice were used for experiments and diabetic mice were generated using the high-fat diet (HFD) low-dose streptozotocin (STZ) protocol as described in our previous publication (9). Briefly, mice, 6 wk of age, were placed on HFD. High-fat diet was from Inotiv Inc. (TD.88137). After 8 wk on HFD, mice were injected with low-dose STZ (40 mg/kg/day, ip, 4 doses on consecutive days) to induce insulin resistance. HFD feeding was then continued for another 4 wk. The oral glucose tolerance test was performed in a few mice in the HFD-STZ group as described previously (10). Mice were euthanized 2 wk after STZ injections and are henceforth described as “diabetic mice.” A timeline of this protocol is shown in Fig. 1*A.* Blood glucose was assessed using Accumeter strips, and plasma insulin was evaluated using a mouse insulin ELISA kit (Crystal Chem Inc.).

### Tissue Preparation

For in vivo experiments, healthy 12-wk-old C57BL/6J mice were fasted for 6 h after which they were given a bolus dose or glucose (2 mg/kg, ip). Following this, mice were humanely euthanized with an overdose of isoflurane anesthesia followed by decapitation after specific time points for artery isolation. For ex vivo experiments, hindlimb (saphenous, popliteal, and gastrocnemius) arteries from fasted mice were collected after euthanasia, cleaned, and immediately placed in high-glucose DMEM (4.5 g/L) with penicillin-streptomycin without FBS at 37°C for specified time points. At the end of the experiments, arteries were removed and placed into ice-cold physiological solution that contained 134 mM NaCl, 6 mM KCl, 2 mM CaCl_2_, 1 mM MgCl_2_, 10 mM Hepes, and 10 mM glucose (pH 7.4) and processed for downstream applications.

### Arterial Electroporation and Protein Knockdown

Plasmid vectors (pGL4.10 luc2, Promega) or small interference RNA (siRNA) sequences targeting either Snail, or scrambled siRNA (scrm) controls (Thermo Fisher Scientific), were inserted into hindlimb arterial segments using low-voltage, square wave electroporator (model CUY21Vivo-SQ, BEX) and the arteries were then maintained in serum-free DMEM-F12 media for 48 h after which they were removed for protein expression or luciferase assays.

### Electrophoretic Mobility Shift Assay

Nuclear extracts from control and diabetic mice hindlimb arteries were prepared using the NE-PER nuclear and cytoplasmic extraction reagents (Cat. No. 78833, Thermo Scientific) following the manufacturer’s instructions and electrophoretic mobility shift assay (EMSA) performed as per standard protocols. Supershift assay was performed using the ChiP-grade Snail antibody (AF3639, Novus Bio.). The protein content of the nuclear extracts was determined using a micro-BCA method (Pierce Biotechnology). Double-stranded oligonucleotides encompassing the E-box sites were labeled with biotin using 3′ end DNA labeling kit (Pierce, Cat. No. 89818) following the supplier’s instructions and used as probes. The sequences of these probes were: -154nt site: 5′- GCGGCTGCAGGTGACCCCGT-3′ and 5′- ACGGGGTCACCTGCAGCCGC-3′; -451nt site: 5′- ACAGAGGCAGGTGGGTCTCT-3′ and 5′- AGAGACCCACCTGCCTCTGT-3′; -698nt site: 5′- TTCTGCACAGGTGGGGCTTA-3′ and 5′- TAAGCCCCACCTGTGCAGAA-3′. Protein-DNA complexes were formed by incubating 5 µg of nuclear extract in a binding buffer [10 mM Tris-HCl, pH 7.5, 50 mM KCl, 1 mM dithiothreitol, 2 µg of poly (dI-dC) and 2.5% glycerol] with 5 nM of biotin-labeled probe in a total volume of 20 µL for 30 min on ice. The protein-DNA complexes were resolved by electrophoresis on a 7.5% polyacrylamide gel using Trisborate-EDTA buffer (44.5 mM Tris-HCl, 44.5 mM borate, and 20 mM EDTA, pH 8.0), transferred to Nylon membrane using the same buffer at 100 V for 1 h, ultraviolet light (UV) cross-linked, and visualized by chemiluminescence. For supershift assay, the reaction mix was incubated with the Snail antibody for 1 h at 4°C before electrophoresis separation. Normal serum was used as a negative control.

### Luciferase Assay

Hindlimb arteries were transfected using electroporation with pGL4.10(luc2) (Promega Corp.) empty vector or vectors containing promoter sequences of either mouse *α-SMA* (+1 to −250) or mouse *TMEM16A* promoter (NM_178642.6, +1 to −750). The promoter sequences were cloned in between EcoRV and HindIII restriction sites (Fig. 3*C*). *TMEM16A* promoter E-box mutations were generated as shown in Fig. 3C and transfected into arteries. Arteries were maintained in serum-free DMEM-F12 media in a regular cell-culture incubator after which they were lyzed and extracts were assayed for luciferase activity using a luciferase assay system (Promega) and a Biotek Synergy plate reader. Raw data were normalized to total protein of each sample. Results are expressed as fold change compared with empty vector transfected.

### Western Blotting

Western blotting for total protein was done following standard protocols. Arterial segments were pooled from 2–3 mice for experiments measuring protein abundance. Proteins were separated on 7.5% SDS-polyacrylamide gels and transferred onto nitrocellulose membranes. Membranes were blocked with 5% nonfat milk and incubated with one of the following primary antibodies overnight at 4°C: anti- TMEM16A (#14476), Snail (#3879), Akt2 (#3063), phospho-protein kinase D1 (#2054), and phospho-GSK3β (Ser9; #5558) were from Cell Signaling, Inc., anti-Ca_V_1.2 (#ab84814) was from Abcam, and anti-actin (#MAB1501) was from MilliporeSigma. Membranes were washed and incubated with horseradish peroxidase-conjugated secondary antibodies at room temperature. Blots were physically cut to allow for probing of multiple proteins without the need for stripping if needed. All primary antibodies were used at 1:500 dilution except anti-actin, which was used at 1:5,000 dilution. We verified that all the primary antibodies used had a minimum of five citations of prior use for detection of the proteins listed here. All secondary antibodies were used at 1:5,000 dilution. Protein bands were imaged using a ChemiDoc gel imaging system (Biorad Inc.), quantified using Quantity One software (Biorad Inc.), and normalized to actin.

### Pressurized Artery Myography

Endothelium intact first-order gastrocnemius muscle arteries were dissected from the hindlimb of mice and cleaned of surrounding tissue. Arterial segments (1–2 mm long) were then cannulated at each end in a perfusion chamber (Living Systems Instrumentation) containing PSS and gassed with 21% O_2_, 5% CO_2_, and 74% N_2_ (pH 7.4) and maintained at 37°C. An attached reservoir was used to alter intravascular pressure and was monitored using a pressure transducer. High glucose (20 mM) PSS was perfused through the chamber and change in arterial diameter was measured at 1 Hz using a CCD camera attached to a Nikon TS100-F microscope and the automatic edge detection function of IonWizard software (Ionoptix, Milton, MA). Myogenic tone was calculated as 100 × (1 − Dactive/Dpassive), where Dactive is active arterial diameter and Dpassive is the diameter determined in the presence of Ca^2+^-free PSS supplemented with 5 mM EGTA.

### Akt Activity Assay

Hindlimb artery lysates were used to measure total Akt activity (Akt Kinase Activity Assay kit, Abcam, ab139436) following manufacturer’s instructions. Arterial lysates were homogenized, incubated with ATP (90 min, 30°C), and the included phospho-specific substrate antibody (60 min, RT) was added in an ELISA plate. After addition of anti-rabbit IgG:HRP conjugate and incubation for 30 min, TMB solution was added and incubated for 60 min. The reaction was stopped using the stop solution provided and absorbance was recorded at 450 nm using a plate reader (Biotek). Values were compared against a standard curve generated by reagents provided with the kit.

### GSK3β Activity Assay

GSK3β activity was measured using a kinase-Glo-based assay (#79700, BPS Bioscience) as per protocol instructions. The 25 µL of a master mix containing 5 µL of 5x kinase assay buffer, 1 µL of ATP (500 µM), 5 µL of GSK substrate peptide (1 mg/mL), and 14 µL of nuclease-free water was first prepared. Whole artery lysate (25 µL) and 25 µL of this master mix were added into each well of a 96-well plate and incubated at 30°C for 45 min. Finally, kinase-Glo Max reagent (50 µL, Promega) was added and incubated for 15 min at room temperature in darkness. The luminescence was then determined by microplate reader (Biotek).

### Statistical Analysis

Statistical analysis was performed using GraphPad InStat and OriginLab. Data are expressed as means ± SE. Student’s *t* test, Mann–Whitney *U* test, and ANOVA with Bonferroni’s post hoc test for multiple group comparisons were used where appropriate. *P* < 0.05 was considered significant. Individual data values for all graphs are provided in the online supplement available at https://doi.org/10.6084/m9.figshare.25284514.v1.

## RESULTS

### High-Fat Diet along with Low-Dose Streptozotocin Induces an Insulin-Resistant, T2D Phenotype in Mice

To achieve a T2D phenotype, mice were placed on a high-fat diet (HFD) followed by four consecutive low doses of streptozotocin (Fig. 1*A*). This treatment increased body weight, such that at 18 wk of age, mice in the HFD-STZ group weighed ∼42 g compared with controls who weighed ∼25 g (Fig. 1*B*). Fasting blood glucose of HFD-STZ mice increased from 11.84 ± 0.44 mmol/L at *week 14*, before STZ injections, to 18.62 ± 0.95 mmol/L at *week 18*, after STZ injections (Fig. 1*C*). In contrast, plasma insulin levels were 73.05 ± 3.85 pmol/L before STZ injections and 58.22 ± 4.02 pmol/L after STZ injections (Fig. 1*D*). At 24 wk of age, oral glucose tolerance test indicated impairment compared with low-fat diet-fed mice (Fig. 1*E*). These data indicate that a high-fat diet combined with STZ leads to obesity, insulin deficiency, and insulin resistance in mice that resembles a T2D phenotype.

### Diabetes Is Associated with a Decrease in the Expression of Snai1 (Snail), a Transcriptional Repressor, in Resistance-Sized Hindlimb Arteries

**Figure 2.**
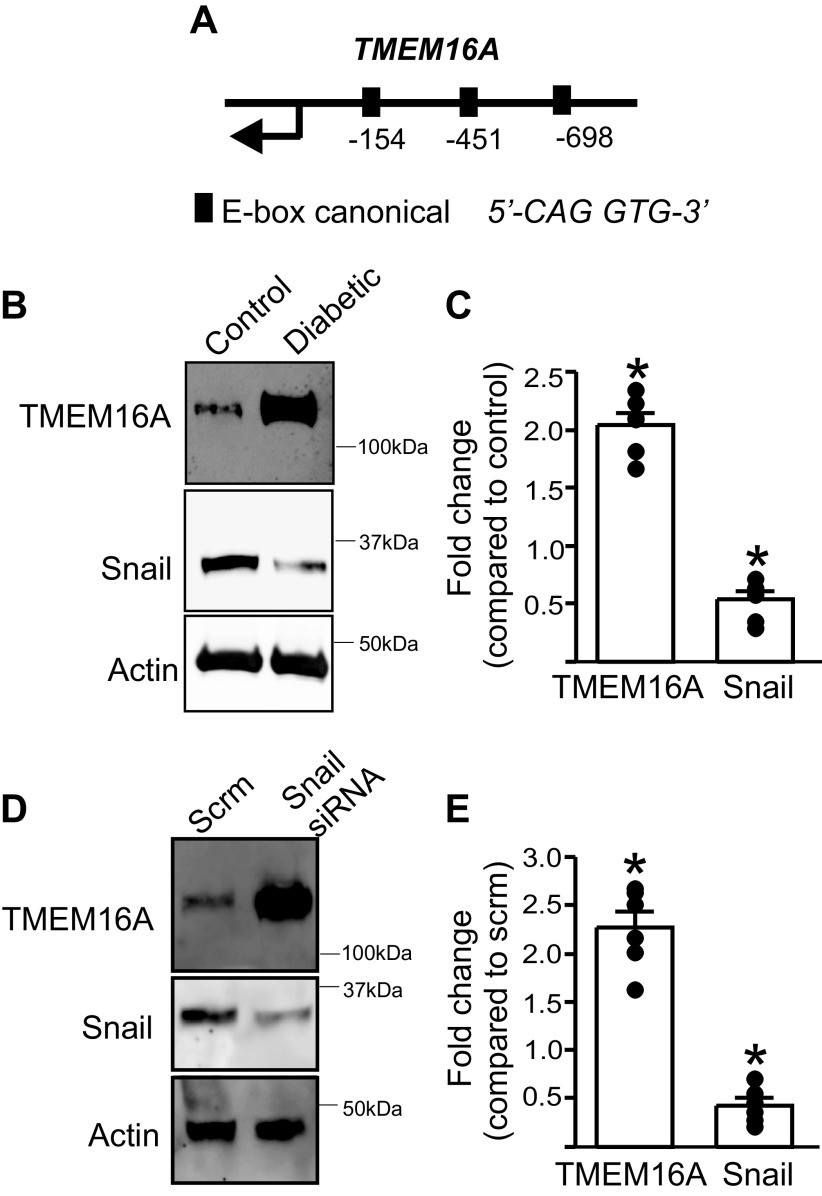
*TMEM16A* gene expression is regulated by its promoter interaction with Snail. *A*: diagram of mouse *TMEM16A* gene showing location of predicted canonical E-box sites (not to scale). *B*: representative Western blot showing TMEM16A and Snail expression in diabetic mice. *C*: mean data. *n* = 5 replicates, *P < 0.05 vs. nondiabetic controls. *D*: representative Western blot showing effect of scrambled (scrm) and Snail siRNA on TMEM16A and Snail expression. *E*: mean data. *n* = 5 replicates, *P < 0.05 vs. respective scrm control. Mann–Whitney *U* test was used for statistical analysis.

To identify transcription factors (TFs) that regulate *TMEM16A* transcription, we used the TF predictor freeware, PROMO (11), to uncover potential TF binding sites in the *TMEM16A* promoter region. The mouse *Anoctamin-1* isoform-1 sequence NM_178642.6 was used as a reference to scan ∼1 kb of genomic region in chromosome 7 upstream of the start codon. We uncovered three consensus “E-box” sequences within the mouse *TMEM16A* promoter (Fig. 2*A*). Results appeared as potential “MyoD” binding sites. MyoD heterodimerizes with other transcription factors to recognize the consensus E-box sequence and these pathways have been extensively investigated (12). A similar analysis of the human *TMEM16A* gene (NM_018043.7) revealed two E-box sites within −1 kb of the start codon. The E-box consensus sequence *-CAG GTG-*, is often bound by “Snail-type” TFs (13, 14). We next probed hindlimb arteries from control and diabetic mice for Snail expression using Western blotting. Results indicated that in resistance size (∼250 µm diameter) hindlimb arteries from diabetic mice, Snail expression was lower than in arteries of control mice (Fig. 2, *B* and *C*). In contrast, TMEM16A channel protein was higher, when compared with those from control mice. Thus, we have identified Snail as a potential transcriptional repressor of *TMEM16A* expression in arteries.

### Snail Knockdown Increases *TMEM16A* Expression in Hindlimb Arteries

We next tested the hypothesis that Snail inhibits *TMEM16A* transcription in arteries. To do this, Snail siRNA was transfected into isolated hindlimb arteries using a low-voltage, square wave electroporator and methods we have previously described (9, 15). Arteries were then maintained in serum-free media for 24 h to enable knockdown, after which protein lysates were collected and Western blotting performed. Results revealed that Snail siRNA reduced Snail protein to 43.7 ± 4.5% of scrambled siRNA controls (Fig. 2, *D* and *E*). Snail knockdown also increased TMEM16A channel expression ∼2.5-fold when compared with scrambled siRNA controls (Fig. 2, *D* and *E*). These results suggest that Snail functions as a transcriptional repressor of *TMEM16A* gene expression.

### Snail Binds to a Consensus E-Box Site in the *TMEM16A* Promoter to Inhibit Transcription

**Figure 3.**
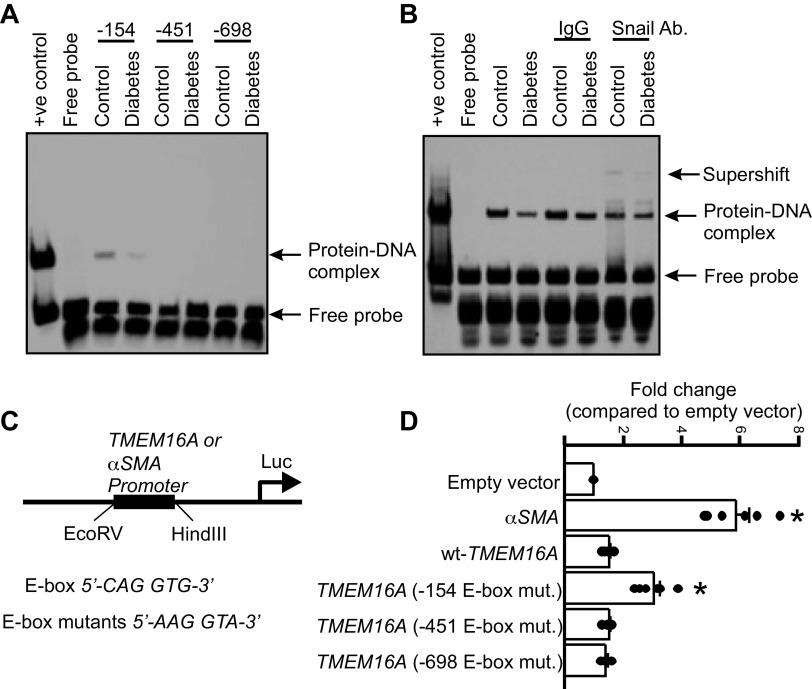
A Snail-mediated repressor complex occupies the *TMEM16A* promoter. *A*: electrophoretic mobility shift assay (EMSA) using biotinylated probes of the corresponding E-box sequences using control and diabetic arterial nuclear extracts. Probe sequences are provided in methods. *B*: supershift assay using the −154-bp sequence probe and a Snail antibody. *C*: diagram showing cloning strategy of the *TMEM16A* or *αSMA* promoter sequences into pGL4.10 vector. The *αSMA* promoter sequence used was +1 to −250 bp of the mouse gene. The *TMEM16A* promoter sequence was +1 to −750 bp of the mouse gene. The *TMEM16A* E-box mutants had their respective sequences mutated as shown in the *D*. Mean luciferase activity after hindlimb arteries were transfected with pGL4.10 vectors containing different promoter sequences. Empty vector was used as negative control while *αSMA* promoter vector was used as positive control for comparison. *n* = 6 replicates, *P < 0.05 vs. empty vector. Mann–Whitney *U* test was used for statistical analysis.

To gain mechanistic insights into the function of Snail at the E-box upstream of the *TMEM16A* promoter, we used an electrophoretic mobility shift assay (EMSA), a qualitative approach to identify specific DNA sequences bound to transcription factors. Biotin-labeled, double-stranded oligonucleotide probes surrounding the DNA sequence of either the −154, −451, or −698 nt E-box sites were incubated with an equal amount of nuclear extract from control and diabetic mouse hindlimb arteries. Protein-DNA binding activity occurred with the −154 bp oligonucleotide in control nuclear extracts that was decreased in lysate from diabetic arteries (Fig. 3*A*). In contrast, oligonucleotides surrounding the −451 and −698 E-box sites did not show any protein-DNA binding (Fig. 3*A*).

Next, we studied the involvement of Snail in protein-DNA binding by performing a supershift assay by using an anti-Snail antibody and the −154 nt oligonucleotide probe. Electrophoresis revealed that Snail was present in the protein-DNA complex from control samples, which was not detectable in diabetic samples (Fig. 3*B*).

We then wanted to test the relative importance of each of the E-box sites in initiating gene transcription. To do this, we selected a nucleotide sequence in the *TMEM16A* promoter that was −750/+1 bp from the start codon, which included the three uncovered E-box sites. This nucleotide sequence was then cloned into a promoterless pGL4.10 (luc2) vector (Fig. 3*C*). Basepair mutations that scrambled the individual E-box sites were also generated in a similar fashion and these luciferase reporter constructs were then transfected into hindlimb arteries using an electroporator. After 48 h of incubation, arterial lysates were collected and analyzed for luciferase activity. A luciferase reporter vector containing the α-smooth muscle actin (SMA) promoter region was used as a positive control to measure expression levels in vascular smooth muscle cells (Fig. 3*D*). Luciferase activity was present in arteries transfected with the α-SMA-pGL4.10 vector, but not in arteries transfected with an empty vector (Fig. 3*D*). In contrast, minimal luciferase activity was observed in control arteries transfected with wt-TMEM16A-pGL4.10 (Fig. 3*D*). Mutation of the −451 and −698 nt E-box sites only marginally affected promoter activity, but mutation of the −154 nt site significantly enhanced promoter activity in control arteries (Fig. 3*D*). These observations indicate that the −154 nt E-box is essential for Snail-mediated repression of the *TMEM16A* promoter. Together, these results suggest that Snail inhibits arterial *TMEM16A* transcription by binding to a consensus E-box sequence located −154 nt upstream from the start codon in the *TMEM16A* promoter region.

### High Extracellular Glucose Stimulates a Transient Increase in *TMEM16A* Expression in Arteries

**Figure 4.**
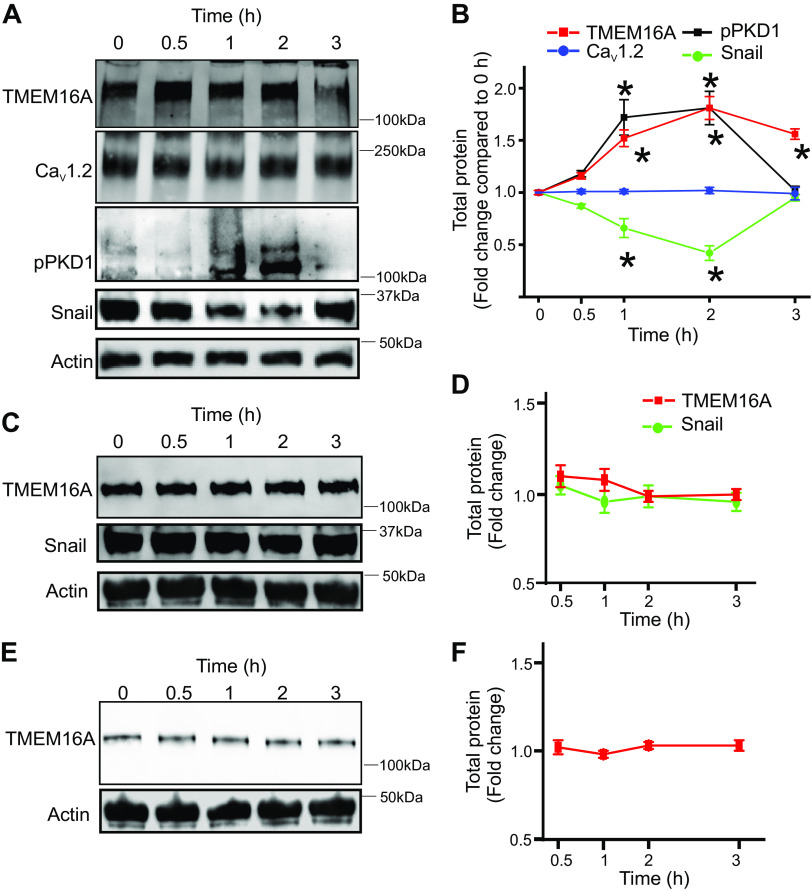
Extracellular glucose affects arterial smooth muscle TMEM16A expression. *A*: representative Western blots of hindlimb arteries for different proteins and at different time points following ex vivo incubation of arteries in high-glucose DMEM media. *B*: mean data of protein expression normalized to actin levels and compared with 0 h. *n* = 5 replicates for each time point, *P < 0.05 vs. 0 h. *C*: representative Western blots from lysate derived from cultured microvascular endothelial cells exposed to high glucose at different time points. *D*: mean data of protein expression normalized to actin levels and compared with 0 h. *n* = 6 replicates for each time point. *E*: representative Western blots from lysate derived from hindlimb arteries exposed to equimolar mannitol in the media. *F*: mean data of protein expression normalized to actin levels and compared with 0 h. *n* = 6 replicates for each time point. Statistical analysis was performed by two-way ANOVA with Bonferroni post hoc test for data in *B, D,* and *F*.

Next, to investigate high glucose and its effect on the Snail-mediated repression of the *TMEM16A* promoter, hindlimb arteries were isolated from nondiabetic mice and incubated in serum and growth factor-free, high-glucose (20 mM) DMEM media for 0.5–3 h at 37°C in a cell-culture incubator after which the arteries were lyzed and protein expression analyzed by Western blotting. Results indicated that TMEM16A expression increased 0.5 h after exposure to 20 mM glucose, an increase that was sustained for ∼2 h (Fig. 4, *A* and *B*). In contrast, glucose reduced Snail expression over the same time course (Fig. 4, *A* and *B*). However, 3 h after high glucose, TMEM16A and Snail expression were not significant from basal levels (Fig. 4, *A* and *B*). In contrast, high glucose did not change Ca_V_1.2 expression (Fig. 4, *A* and *B*). To verify if the change in TMEM16A and Snail expression occurred only in smooth muscle cells, cultured mouse microvascular endothelial cells were treated with a similar high-glucose protocol. Results indicated that TMEM16A and Snail expression were unchanged in endothelial cells exposed to brief high-glucose stimulation (Fig. 4, *C* and *D*). High glucose increases the osmolarity of the media, which could influence TMEM16A expression. To rule out expression changes due to changing osmolarity, arteries were incubated for similar time points in media that contained equimolar mannitol. Results from these experiments indicate that changes in osmolarity do not affect TMEM16A expression (Fig. 4, *E* and *F*). Together, these results suggest that acute high glucose exposure decreased Snail but increased TMEM16A expression in arterial smooth muscle cells treated with high glucose ex vivo.

### High Glucose Stimulates Acute Activation of Protein Kinase D1

As the transient increase in TMEM16A expression occurred in the absence of growth factors, it was likely that the pathway was not dependent on insulin-receptor signaling. We hypothesized that it might be a direct effect of high extracellular glucose on smooth muscle cell-signaling pathways. High glucose is known to activate several isoforms of protein kinase C that could lead to the activation of protein kinase D1, which could phosphorylate Snail and accelerate its degradation. We probed our time course blots for phospho-protein kinase D1, the activated form of enzyme. Results indicated that high glucose increased phospho-protein kinase D1, which correlated with a decrease in Snail and an increase in TMEM16A expression (Fig. 4, *A* and *B*). These data suggest that high extracellular glucose likely directly activates protein kinase D1 to disrupt the Snail-mediated repression of the *TMEM16A* promoter.

### High Glucose Causes Transient Vasoconstriction in Nondiabetic Arteries

**Figure 5.**
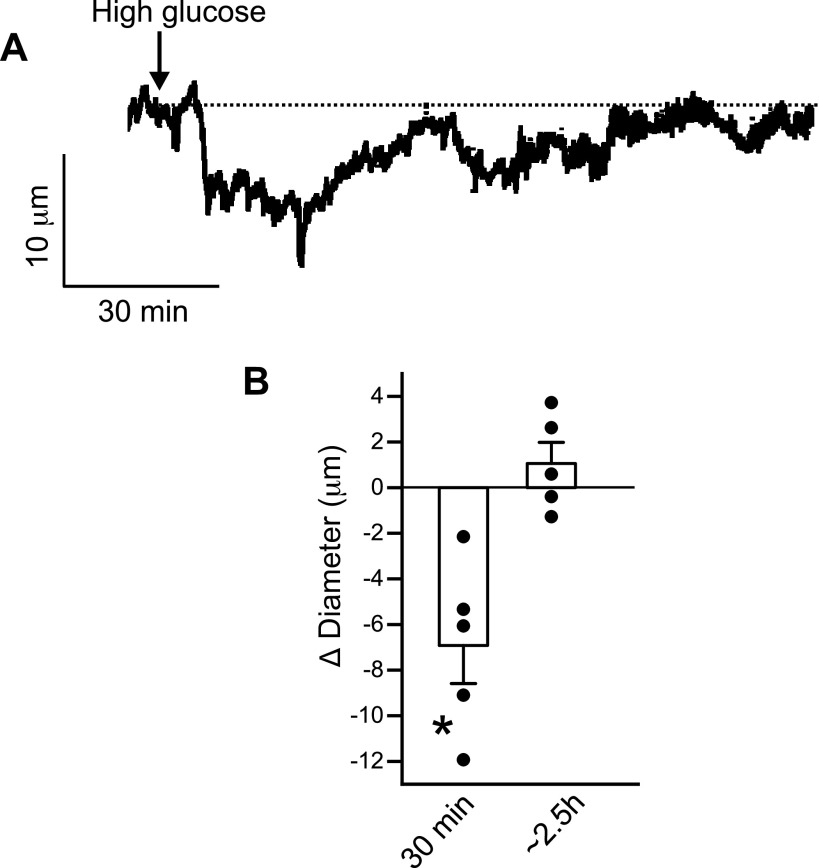
High glucose induces a transient constriction in pressurized hindlimb arteries. *A*: raw traces of nondiabetic hindlimb artery when perfused with 20 mM high glucose PSS. *B*: mean Δ diameter (µm) after 30 min and ∼2.5 h of high glucose perfusion, *n* = 5 replicates for each, *P < 0.05 vs. baseline value before glucose administration. Statistical analysis was performed using a one-way ANOVA with Bonferroni post hoc test for data in *B*.

The effect of high glucose on the contractility of hindlimb arteries was then measured using pressurized artery myography. Gastrocnemius muscle arteries from nondiabetic mice were cannulated and pressure-induced vasoconstriction was measured. Results indicated that in hindlimb arteries, perfusion of high glucose induced a rapid constriction of ∼7 µm, followed by slow recovery to baseline levels in ∼2.5 h (Fig. 5, *A* and *B*). These data indicate that high glucose triggers transient vasoconstriction in mouse hindlimb arteries.

### An Increase in Blood Glucose Stimulates Acute Activation of Protein Kinase D1 to Increase TMEM16A Expression in Nondiabetic Mice

**Figure 6.**
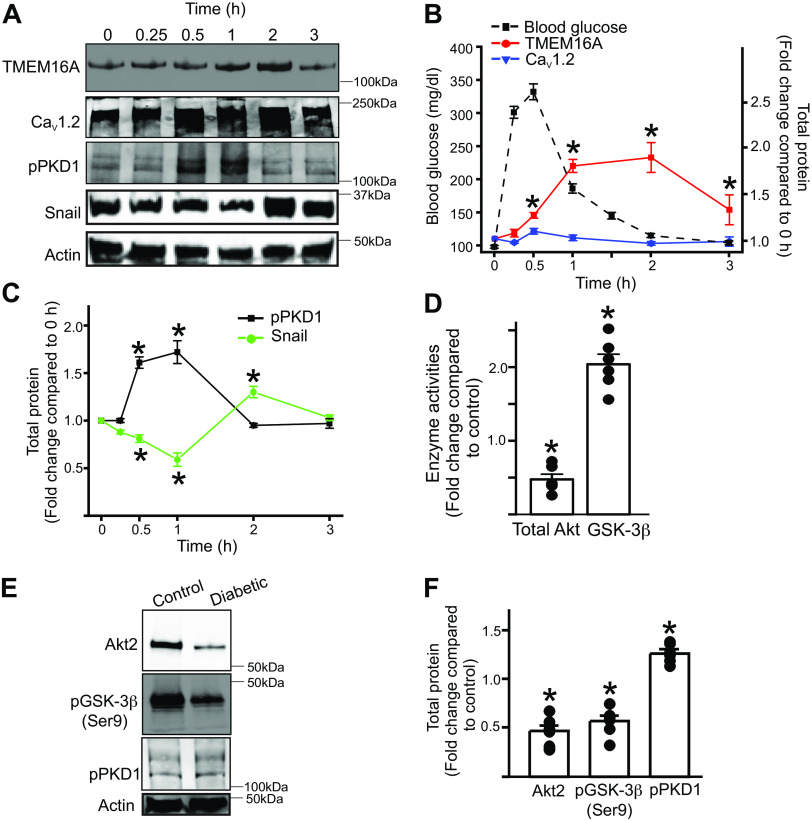
Protein kinase D1 and GSK3β independently upregulate TMEM16A expression. *A*: representative Western blots of hindlimb arteries at different time point following in vivo injection of bolus dose of glucose to fasted control mice. *B*: mean data of different protein expression in relation to blood glucose. *n* = 5 replicates for each time point, *P < 0.05 vs. 0 h. *C*: mean data of hindlimb pPKD1 and Snail expression after glucose injections. *n* = 5 replicates for each time point, *P < 0.05 vs. 0 h. *D*: mean diabetic artery total Akt and GSK3β enzyme activities compared with nondiabetic controls. *n* = 5 replicates for each, *P < 0.05 vs. nondiabetic control. *E*: representative Western blots comparing hindlimb protein expression from control and diabetic mice. *F*: mean data, *n* = 5 replicates for each, *P < 0.05 vs. nondiabetic control. Statistical analysis was performed using a two-way ANOVA with Bonferroni post hoc test for data in *B* and *C*, and Mann–Whitney *U* test for *D* and *F*.

Next, we investigated the in vivo effect of glucose on TMEM16A channel expression in arteries of nondiabetic mice. To investigate this, control mice were fasted for 6 h after which they were given an intraperitoneal bolus dose of glucose (2 g/kg). Hindlimb arteries were collected at *time 0* (no treatment) or from the mice up to 3 h after glucose injections. Plasma glucose levels were analyzed using AlphaTrak2 test strips. Data indicate that plasma glucose concentrations increased rapidly after glucose injections, followed by a gradual decline to baseline 3 h later (Fig. 6*B*). Western blotting indicated that arterial TMEM16A channel protein increased at 0.5 h, peaked at 2 h, and gradually returned to fasting levels 3 h after glucose administration (Fig. 6, *A* and *B*). Two hours after glucose administration, TMEM16A protein was approximately twofold higher than control (Fig. 6*B*). Phospho-protein kinase D1 levels also increased over the same time course, whereas Snail expression correspondingly decreased. The expression of all these proteins returned to basal levels 3 h after glucose administration (Fig. 6, *A* and *C*). In contrast, Ca_V_1.2 expression did not change over the same period (Fig. 6, *A* and *B*). These results suggest that in nondiabetic control mice, an increase in blood glucose triggers a protein kinase D1-mediated downregulation of Snail, which likely then activates *TMEM16A* transcription.

### Dysfunctional Akt Signaling Leads to Activation of GSK3β, which Negates Snail-Mediated Repression of the *TMEM16A* Promoter during Diabetes

We previously reported that dysfunctional Akt signaling increases TMEM16A expression in arterial smooth muscle cells during diabetes (9). Insulin resistance leads to dysfunctional Akt signaling and activation of GSK3β. GSK3β and protein kinase D1 can each independently phosphorylate Snail leading to its removal from the nucleus (16–18). Hence, we next investigated if the expression and activities of GSK3β and protein kinase D1 were altered in arteries during diabetes. Enzyme activity assays revealed that total Akt activity was lower, whereas GSK3β activity was higher in arteries of diabetic mice (Fig. 6*D*). Western blotting indicated that both Akt2 expression and phospho-GSK3β (ser9), the inactive form of GSK3β, were decreased during diabetes (Fig. 6, *E* and *F*). In contrast, there was only a small increase in phospho-protein kinase D1 levels in diabetic arteries (Fig. 6, *E* and *F*). These data indicate that GSK3β was active and that protein kinase D1 possibly had a smaller role in Snail regulation and long-term upregulation of TMEM16A expression in diabetic arteries. Together, these results suggest that dysfunctional Akt signaling activates GSK3β, negating the repressor effect of Snail on the *TMEM16A* promoter to activate its gene transcription in arteries of diabetic, insulin-resistant mice.

In summary, these data reveal that Snail, a transcriptional repressor, is constitutively positioned at an E-box site on the *TMEM16A* promoter in arterial smooth muscle. High extracellular glucose and/or dysfunctional Akt signaling acting via protein kinase D1 and GSK3β, respectively, phosphorylate and remove Snail from the promoter to initiate *TMEM16A* gene transcription increasing protein expression and triggering vasoconstriction.

## DISCUSSION

Here, we demonstrate that arterial *TMEM16A* gene transcription is regulated by a Snail-mediated repressor complex. In hindlimb arteries from control mice, transient increase in extracellular glucose activates protein kinase D1, which phosphorylates Snail causing it to be removed from the *TMEM16A* promoter thereby activating gene transcription. Similarly, in hindlimb arteries from diabetic mice, we found that a decrease in Akt activity activated GSK3β, which induced phosphorylation and removal of Snail from the *TMEM16A* promoter to increase transcription. These data highlight the Snail-mediated regulation of *TMEM16A* transcription and the differential modulation of TMEM16A expression in control and diabetic arteries to modulate arterial contractility.

We had shown previously that T2D leads to increased arterial smooth muscle TMEM16A protein, currents, and hindlimb artery myogenic tone, leading to increased vasoconstriction (9). We demonstrated that the upregulation of TMEM16A in diabetic arteries was linked to downregulation of Akt2 expression and activity (9). Drawing upon this earlier observation, we proceeded to investigate the molecular pathways that regulated arterial *TMEM16A* gene transcription. We first screened the mouse *TMEM16A* promoter region starting from +50 nt to −1,000 nt from the start codon. This genomic region in mouse chromosome 7 was found to contain three canonical “E-box” sites. An E-box (enhancer box or “DNA response element”) is a collective term for nucleotide sequences with a consensus sequence *-CAN NTG-*, where *“N”* can be any nucleotide (19, 20). These sequences are often found in gene promoters and can act as protein-binding sites for various transcription factors (TFs) and their protein complexes to affect gene expression (19). Proteins that bind to this motif are of the basic helix-loop-helix (bHLH) or the “zinc-finger” class of TFs with binding specificity depending on nucleotide substitutions in the *“N”* position (21). The bHLH group of TFs can be activators or repressors of gene transcription, whereas most members of the zinc-finger family of TFs act as transcriptional repressors (19, 20, 22).

As TMEM16A expression was upregulated in diabetic hindlimb arteries, we proceeded to investigate if a transcriptional repressor was involved in arterial *TMEM16A* expression. The E-box sites that we uncovered in the *TMEM16A* promoter at −154, −451, and −698 nt were of the sequence *-CAG GTG-*, which is suggested to be the binding site for “Snail-type” zinc-finger transcriptional repressors that include Snail (Snai1), Slug (Snai2), and Sip1 (Smad interacting protein 1) (13, 14). Here, in diabetic and in Snail-specific siRNA-transfected control arteries, expression of Snail decreased while TMEM16A increased, which suggested a possible regulatory function for Snail over *TMEM16A* expression. Snail-mediated repression of an ion channel gene has not been described before. In mammals, transcriptional repression by Snail has been studied in early embryonic development and during epithelial-to-mesenchymal transition (EMT). Specifically, Snail inhibits expression of E-cadherin, a key adherens junction protein, by binding to E-boxes in the gene’s promoter, thereby inducing EMT (23, 24). Snail has also been shown to directly repress its own gene transcription (25) and that of mucin-1 (MUC-1) (26), proteins of the tight junctions including claudins and occludin (27), and the β-subunit of the Na^+^, K^+^ ATPase (28). While such previous studies highlight the role of Snail in tumor progression, the data shown here provide evidence that Snail could also be involved in the progression of diabetic vascular disease.

To investigate if any of the identified E-box sites in the *TMEM16A* promoter bound to and mediated the Snail-induced repression of the *TMEM16A* promoter, we used oligonucleotide probes corresponding to the E-box regions and electrophoretic mobility shift assays to analyze DNA-protein binding. Interestingly, we only observed DNA-protein binding with the probe surrounding the −154 nt region and not with the other two identified E-box regions. The presence of Snail in the protein complex bound to the DNA probe was further confirmed by using an anti-Snail antibody and performing a supershift assay. These results suggest that a Snail-mediated protein complex bind to the −154 nt sequence in the *TMEM16A* promoter. Next, to assess the role of Snail in altering *TMEM16A* promoter activity, we cloned a 750-bp region of the promoter into a luciferase expression plasmid, transfected it into intact hindlimb arteries, and probed the tissue lysate for luciferase activity. The “wildtype” sequence had all its E-box sites intact, while we also tested vectors that contained specific mutated E-box regions. Snail binding to an E-box site in the cloned promoter region would decrease promoter activity and prevent luciferase expression. Results revealed that only mutation of the −154 nt E-box significantly increased promoter activity, which suggested that the −154 nt was the primary E-box site that was bound by Snail. Together, these data suggest that a consensus E-box sequence located at −154 nt from the start codon of the *TMEM16A* gene harbors a Snail-mediated repressor complex that constitutively prevents *TMEM16A* transcription in arteries. While it is possible that other closely associated repressors and Snail-interacting partners like Slug and Sip1 could also be present in this protein complex, we did not investigate them further.

We then investigated which signaling mechanisms alter the Snail-repressor complex occupancy of the *TMEM16A* promoter. In healthy, nondiabetic individuals, an increase in blood glucose, as would occur after a meal, is followed by an immediate increase in insulin levels. Insulin binds to the insulin receptor, leading to downstream Akt phosphorylation, which in turn phosphorylates and deactivates GSK3β (29, 30). Basal Akt activity is essential for regulating GSK3β function (29, 30). T2D, in contrast, is a disease associated with an increase in circulating blood glucose and dysfunctional insulin receptor signaling leading to decreased Akt activity (31–33). GSK3β can phosphorylate Snail inducing its migration from the nucleus and subsequent proteasomal degradation (34–36). In our studies, we found that in diabetic arteries, there was downregulation of total Akt activity while GSK3β activity increased and p-GSK3β expression decreased. These data suggest that in diabetic arteries, an upregulation of GSK3β activity induces a downregulation of Snail and an increase in TMEM16A expression. Although this mechanism would explain downregulation of Snail in diabetic arteries, it did not account for the loss of arterial Snail during physiological hyperglycemia in healthy animals.

In experiments in nondiabetic animals, an increase in blood glucose decreased Snail expression while increasing TMEM16A expression, which reversed as blood glucose levels returned to baseline. These results suggest that the Snail is quickly removed from the promoter when blood glucose levels rise to initiate *TMEM16A* transcription. However, ex vivo experiments where isolated arteries were incubated in serum- and growth factor-free high-glucose DMEM media also showed similar results. These ex vivo results were surprising given that unlike in vivo where blood glucose levels physiologically return to baseline after 3 h, extracellular glucose levels ex vivo did not change and remained constant throughout the course of the experiment. High glucose-induced changes in osmolarity were ruled out by experiments where the osmolarity of the media was adjusted using equimolar mannitol. High glucose also showed no effect on TMEM16A expression in cultured endothelial cells. Thus, the results from the in vivo and ex vivo high-glucose experiments suggest that in control arteries, *1*) change in Snail and TMEM16A expression was not due to insulin-receptor signaling and was likely a direct effect of high glucose-initiated signaling pathways in smooth muscle cells and *2*) in control arteries, cellular signaling pathways initiated by prolonged high extracellular glucose likely desensitize to return Snail and TMEM16A expression to basal levels.

High extracellular glucose is known to directly activate several protein kinase C isoforms (32). Protein kinase D1 was first classified as protein kinase C µ (PKCµ) as it shared certain similarities with other protein kinase C isoforms (37), but distinct structural and enzymatic properties led to it being reclassified separately (37, 38). Protein kinase D1 is activated by several PKCs and apart from GSK3β is the only other known kinase that can mediate Snail phosphorylation and removal from the nucleus (16, 17, 37, 39). Results from both the in vivo and ex vivo high-glucose challenge revealed that hindlimb artery phospho-protein kinase D1 expression increased while Snail expression decreased, which suggests that high glucose triggers protein kinase D1-induced Snail phosphorylation. Interestingly, although glucose levels remained elevated throughout the course of the ex vivo experiments, protein kinase D1, Snail, and TMEM16A expression levels were similar to what was observed in the in vivo experiments. These results suggest that high glucose induces a brief upregulation of TMEM16A expression and activity, but prolonged exposure of nondiabetic arteries desensitizes the protein kinase D1-mediated upregulation of TMEM16A expression.

These data were further verified with the pressurized artery experiments, where even in the continuous presence of high glucose, constriction in nondiabetic arteries gradually returned to baseline after ∼3 h. We also found that hindlimb artery Ca_V_1.2 expression did not change with either ex vivo or in vivo high-glucose challenge. These results were consistent with previous reports that found no change in Ca_V_1.2 expression but increased channel activity in arterial myocytes after high-glucose treatment (7, 40, 41). Prior work from our group had also shown that TMEM16A is activated by (Ca^2+^)_i_, including Ca^2+^ signals arising from Ca_V_1.2 and TRP channels (42–44). Hence, it is likely that high extracellular glucose triggers synergistic activation of both Ca_V_1.2 and TMEM16A to induce arterial constriction. However, the precise physiological role of the observed high glucose-induced transient increase in arterial smooth muscle TMEM16A expression to induce vasoconstriction remains unclear. It is possible that this response serves to prevent a postprandial glucose surge into peripheral organs and tissue.

Further experimentation with diabetic arteries here showed that Akt2 expression and total Akt activity were significantly decreased. Downstream to this, GSK3β (pSer9), the inactive form of GSK3β, decreased, whereas GSK3β activity increased. Phospho-protein kinase D1, however, was only slightly increased. These results suggest that long-term potentiation of TMEM16A expression in diabetic arteries is primarily due to activation of GSK3β. In T2D, metabolic syndrome and insulin resistance lead to dysfunctional insulin receptor signaling, which in turn affects downstream Akt signaling (31–33). Akt inhibits GSK3β and GSK3β knockout mice are resistant to the development of T2D (45–47). The role of GSK3β in the development of diabetic vascular dysfunction and its potential as a drug target is well known (46, 48, 49). However, the function of protein kinase D1 in different tissues is still unclear. Protein kinase D1 is reported to be essential for insulin secretion from pancreatic β cells (50, 51), whereas, in adipocytes, gene deletion improved insulin sensitivity and liver steatosis (52). In cardiomyocytes, protein kinase D1 overexpression improved insulin resistance (53), but in contrast, loss in activity preserved cardiac function in obesity (54). It is now suggested that protein kinase D1 activity can either be protective or deleterious depending on the metabolic state of the heart (55). Overall, our results indicate that in T2D arteries, while direct effects of high glucose on protein kinase D1 signaling could still be involved, GSK3β signaling is the primary pathway that causes upregulation of arterial TMEM16A expression.

In conclusion, here we demonstrate that arterial *TMEM16A* transcription is regulated by a Snail-mediated repressor complex and that high glucose and insulin resistance activate protein kinase D1 and GSK3β, respectively, to induce phosphorylation and degradation of Snail to increase TMEM16A expression in arterial smooth muscle and cause vasoconstriction.

## DATA AVAILABILITY

Individual data points are shown in the figures where possible. All data points used to generate the main figure bar graphs are available at figshare TMEM16A dataset: https://doi.org/10.6084/m9.figshare.25284514.v1.

## SUPPLEMENTAL DATA

10.6084/m9.figshare.25284514.v1 Supplemental material: https://doi.org/10.6084/m9.figshare.25284514.v1.

## GRANTS

This research was funded by the National Institutes of Health R01 grants, HL149662 to M.D.L and R01 HL155180, HL158846, and HL166411 to J.H.J.

## DISCLOSURES

No conflicts of interest, financial or otherwise, are declared by the authors.

## AUTHOR CONTRIBUTIONS

J.H.J. and M.D.L. conceived and designed research; S.R., M.A.B., A.B., A.M-D., and M.D.L. performed experiments; S.R., M.A.B., A.B., A.M-D., and M.D.L. analyzed data; S.R., M.A.B., A.B., A.M-D., J.H.J., and M.D.L. interpreted results of experiments; S.R., M.A.B., and M.D.L. prepared figures; M.D.L. drafted manuscript; J.H.J. and M.D.L. edited and revised manuscript; J.H.J. and M.D.L. approved final version of manuscript.
